# Lipid Droplet-Related PLIN2 in CD68^+^ Tumor-Associated Macrophage of Oral Squamous Cell Carcinoma: Implications for Cancer Prognosis and Immunotherapy

**DOI:** 10.3389/fonc.2022.824235

**Published:** 2022-03-15

**Authors:** Yijia He, Yuexin Dong, Xinwen Zhang, Zhuang Ding, Yuxian Song, Xiaofeng Huang, Sheng Chen, Zhiyong Wang, Yanhong Ni, Liang Ding

**Affiliations:** ^1^ Central Laboratory of Stomatology, Nanjing Stomatological Hospital, Medical School of Nanjing University, Nanjing, China; ^2^ Department of Oral Pathology, Nanjing Stomatological Hospital, Medical School of Nanjing University, Nanjing, China

**Keywords:** PLIN2, oral squamous cell carcinoma, prognosis, tumor-associated macrophage, immune checkpoint

## Abstract

**Background:**

PLIN2 (adipose differentiation-related protein) belongs to the perilipin family and is a marker of lipid droplets (LDs). Numerous types of tumor exhibit a high PLIN2 level, but its tumorigenic or tumor-suppressive role has been in debate. Recently, LDs serve as innate immune hubs and show antimicrobial capacity. We here aimed to investigate the heterogeneous functions of PLIN2 in the tumor microenvironment and immune regulation.

**Methods:**

This retrospective study included 96 oral squamous cell carcinoma (OSCC) samples and analyzed the spatial distribution of PLIN2 by immunohistochemistry (IHC) and LD level by oil red O staining. A total of 21 serial sections were obtained to analyze the relationship between PLIN2 and immune cells by IHC and immunofluorescence (IF). Single-cell sequencing was used to analyze the cell locations of *PLIN2*. The values of diagnosis and prognosis of PLIN2 were also evaluated. Tumor Immune Estimation Resource (TIMER), cBioPortal databases, and IHC analysis were used to investigate the relationship between PLIN2 and OSCC immune microenvironment.

**Results:**

PLIN2 was mainly expressed in tumor-infiltrating immunocytes (TIIs) of OSCC. Patients with high PLIN2 harbored more cytoplastic LDs. CD68^+^ tumor-associated macrophages (TAMs), instead of T cells and B cells, were found to be the main resource of PLIN2 in OSCC stroma and lung, pancreas, prostate, and testis. However, CD56^+^ NK cells also showed less extent of PLIN2 staining in OSCC. Moreover, patients with a high PLIN2 level in immune cells had a higher TNM stage and were susceptible to postoperative metastasis, but the escalated PLIN2 level in invasive tumor front independently predicted shorter metastasis-free survival. Furthermore, a high PLIN2 presentation in the microenvironment induced immune suppression which was featured as less infiltration of CD8^+^ T cells and more CD68^+^ TAMs and Foxp3^+^ Tregs, accompanied by more immune checkpoint molecules such as *CSF1R*, *LGALS9*, *IL-10*, *CTLA-4*, and *TIGIT*.

**Conclusion:**

CD68^+^ TAM-derived PLIN2 might participate in regulating immune balance of OSCC patients, which provides new insight into immune checkpoint therapy.

## Introduction

Oral squamous cell carcinoma (OSCC) is a common malignant tumor of the head and neck region, whose 5-year overall survival rate is approximately 50% (1). The deadliest aspect of OSCC tends to be cervical lymph node metastasis because the lymphatic vessels are abundant and comprise numerous anastomoses in the oral cavity (2, 3). Therefore, the discovery of specific predictors and new cancer therapy is critical for improving the prognosis of OSCC patients. *PLIN2* is upregulated in parallel with lipid storage during lipid droplet (LD) formation and exists on the surface of LDs from the earliest time of their synthesis (4). Furthermore, the reduction of PLIN2 would allow for lipolysis to increase the availability of energy precursors to support cell metabolism (5). Specifically, ATP is generated through mitochondrial respiration to support cell proliferation and the increased reliance on glycolysis is a known sign of cancer biology (6). Moreover, PLIN2 can be expressed in various tissues such as lactating mammary gland, liver, lung, and skeletal muscle, reflecting the severity of lipid droplet-related diseases, including atherosclerosis and fatty liver (7–9). Notably, Mrozinski et al. noted the strong expression of PLIN2 in lipid-laden macrophages (7).

It has been well recognized that lipogenic metabolism may be altered in various types of tumors, which is a key feature of sustained cellular proliferation in tumor biology. In clear cell renal cell carcinoma (ccRCC), PLIN2 was elevated in tumors and correlated with HIF-2α (10). In gastric carcinoma, the neoplastic cells with PLIN2 overexpression obtained more conspicuously rapid growth *in vivo* (11). In cutaneous melanomas, high PLIN2 expression was associated with poor metastasis-free survival (MFS), disease-free survival (DFS), and overall survival (OS) rates of patients (12). However, in uterine leiomyoma, PLIN2 deficiency reprogrammed cells to a hyperproliferation phenotype (13). Therefore, PLIN2 exerts both tumorigenic and tumor suppressive roles during tumor development in a tumor content-dependent manner.

Recently, Bosch et al. discovered that various proteins on LDs take part in innate immunity in response to bacterial lipopolysaccharide (14). A study reported that the numbers and protein composition of LDs can be changed by myeloid cell activation (15). Moreover, research has shown that LD accumulation may be one of the causes of EGFR-TKI resistance in lung cancer (16). PLIN2, as a marker of LDs, was found to increase the expression of pro-inflammatory cytokines in THP-1 macrophages (17). However, its effect in the modulation of immune cell function remains to be further explored.

Therefore, in our study, we examined the expression pattern and clinical diagnostic and prognostic value of PLIN2 protein in the tumor microenvironment (TME) including tumor cells (TCs), fibroblast-like cells (FLCs), and tumor-infiltrating immunocytes (TIIs). Moreover, we investigated the co-localization of immune cell markers and PLIN2 in OSCC by immunohistochemistry (IHC) and other tissues by a database of single-cell sequence. Importantly, relationships between PLIN2 and immune balance and immune checkpoint pathways were also examined.

## Materials and Methods

### Patients and Samples

All methods used for this study were approved by the Ethics Committee of Nanjing Stomatology Hospital (2019NL-009(KS)). The study was carried out in accordance with the Declaration of Helsinki. Written informed consent was obtained from all the patients. From 2005 to 2017, 96 primary OSCC patients were enrolled. The inclusion and exclusion criteria of patients were the same as those of our previous studies (18). These patients were followed up for 2–60 months, and the median was 39 months. Paraffin-embedded OSCC tissue slices were obtained from the pathology department and used for IHC study.

### Immunohistochemistry and Quantification

The protocol of IHC of formalin-fixed paraffin-embedded sections was performed as previously described (19). Anti-PLIN2 (ab181452, Abcam, Waltham, MA, USA) was used at a dilution ratio of 1:300, and the serial sections were incubated with primary antibodies such as anti-CD4 (ZSGB-BIO, ZM-0418), anti-CD8 (ZSGB-BIO, ZA-0508), anti-CD19 (ZSGB-BIO, ZM-0038), anti-CD56 (ZSGB-BIO, ZM-0057), anti-CD68 (ZSGB-BIO, ZM-0464), and anti-FOXP3 (ab253297, Abcam). We used PBS to replace the primary antibody as negative control. The IHC staining results of PLIN2 was assessed by ImageJ software (20, 21). Digital photos were acquired from the tumor center and invasive front, and the average values were calculated for further analysis (22).

### Gene Correlation Analysis in cBioPortal

The cBioPortal for Cancer Genomics (http://cbioportal.org) is a website for exploration of multidimensional cancer genomics data, providing readily understandable gene expression events (23). We used cBioPortal to analyze the correlation between *PLIN2* and specific immune cell subset markers as well as specific immune checkpoint molecules in head and neck squamous cell cancer (HNSCC). Co-expression was calculated based on the cBioPortal’s online instructions.

### RNA Sequencing and Analysis

The Cell Type Atlas showed that single-cell RNA sequencing (scRNAseq) analysis was based on publicly available genome-wide expression data (GSE164690 and https://www.proteinatlas.org/ENSG00000147872-PLIN2/celltype). These clusters have been annotated as 51 cell types using >500 well-known cell type-specific markers. The genes expressed in each of the cell types can be explored in interactive UMAP plots and bar charts, with links to corresponding immunohistochemical staining in human tissues.

### Immunofluorescence

Frozen tumor tissue sections were fixed with paraformaldehyde, washed three times with PBS, and blocked with 3% bovine serum albumin (BSA, G5001, Servicebio, Wuhan, China) to block non-specific binding for 30 min. Then, the tissue sections were incubated with primary antibodies against PLIN2(ab181452, Abcam) and CD68 (977785, CST) overnight at 4°C, followed by secondary antibodies(Servicebio). After washing, DAPI (G1012, Servicebio) was stained and coverslips were mounted with ProLong Gold Antifade Reagent (Invitrogen, Carlsbad, CA, USA). Images were acquired with a Zeiss Axio Observer Z1 microscope (Carl Zeiss, Jena, Germany).

### Oil Red O Staining

Frozen slices were fixed in the fixative solution for 15 min, then rinsed 3 times in 1× PBS. Slides were then placed in oil red O (ORO) solution for 10 min in the dark and incubated in 60% isopropanol for 30 s. The tissue sections were counterstained with hematoxylin for 5 min and, finally washed twice, photographed, and counted.

### The Human Protein Atlas

The Human Protein Atlas (https://www.proteinatlas.org/) is a Swedish-based program initiated in 2003 with the aim at mapping all the human proteins in cells, tissues, and organs using an integration of various omics technologies, including antibody-based imaging, mass spectrometry-based proteomics, transcriptomics, and systems biology. All the data in the knowledge resource were open access to allow scientists both in academia and in industry to freely access the data for exploration of the human proteome. Single-cell RNA in cell types from tissue can be shown separately for each analyzed tissue; we used the human protein atlas to study the expression pattern of *PLIN2* in different tissues.

### TIMER Analysis

TIMER (https://cwastrome.shinyapps.io/timer/) is a user-friendly website for cancer researchers to evaluate the comprehensive correlation analysis between TII markers and selected genes. We used TIMER to assess the correlation between *PLIN2* and markers of specific immune infiltrating cell subset at transcription level.

### Statistical Analysis

SPSS 18.0 and GraphPad Prism 8.0 software packages were used for data analysis and graphic processing. Pearson’s chi-square test was used to compare clinicopathological features. The Mann–Whitney U test was used to compare the two groups. Survival analysis includes OS, MFS, and DFS, which were evaluated by the Kaplan–Meier and log-rank tests. Further multivariate analysis was carried out by the Cox proportional hazards regression model to determine the independent risk factors, adjusted hazard ratio (HR), and 95% confidence interval (CI) of OSCC. Co-expression between PLIN2 and immune checkpoint molecules was investigated by Pearson correlation analysis. All statistical tests were two-sided, and p < 0.05 was considered to be significant.

## Results

### Tumor-Infiltrating Immunocytes Are the Main Resource of PLIN2 in the OSCC Microenvironment

**Figure 1:**
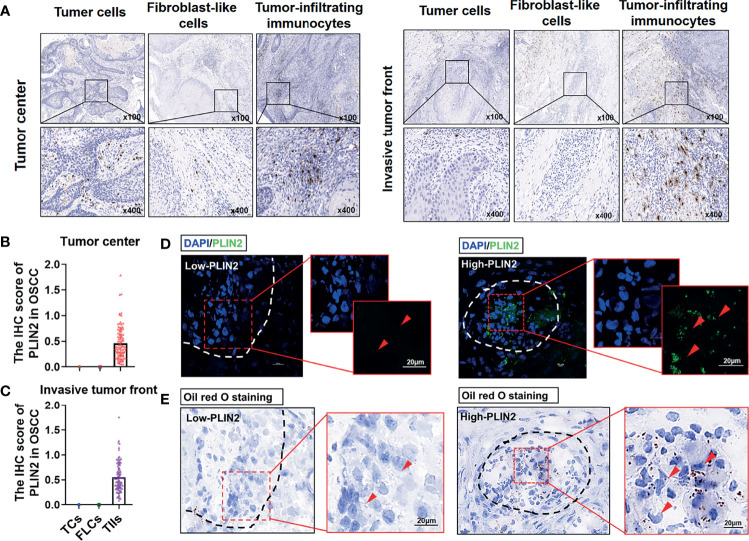
The distribution of PLIN2 in OSCC tissue samples. Representative immunohistochemistry staining of PLIN2 in tumor center and invasive tumor front **(A)**. The IHC score of PLIN2 in TCs, FLCs, and TIIs from OSCC patients in tumor center and invasive tumor front **(B, C)**. Immunofluorescent was performed to detect the PLIN2 levels in OSCC tissues. The bar is 20 μm **(D)**. Oil red O staining was performed to examine LD accumulation **(E)**.

The results showed positive staining for PLIN2 in 96 OSCC samples. As shown in **Figure 1A**, both in the tumor center and invasive tumor front, the expression of PLIN2 in OSCC sections had a cytoplasmic and membranous expression and mostly granular staining pattern. Moreover, PLIN2 was positively presented in TIIs but negatively expressed in TCs and FLCs. PLIN2 in TIIs had higher IHC scores (**Figures 1B, C**). Furthermore, to clarify the regulation of lipid metabolism mediated by PLIN2, oil red O staining assay and IF were conducted as visual indicators of intracellular lipids in OSCC. The results showed that LDs were obviously decreased with low-expressing PLIN2, while a significant increase in LD deposits was observed in OSCC tissues with overexpressing PLIN2 (**Figures 1D, E**), indicating that PLIN2 was mainly expressed in TIIs and positively correlated with LD content in OSCC tissues.

### CD68^+^ Tumor-Associated Macrophages Show a High PLIN2 Expression

**Figure 2:**
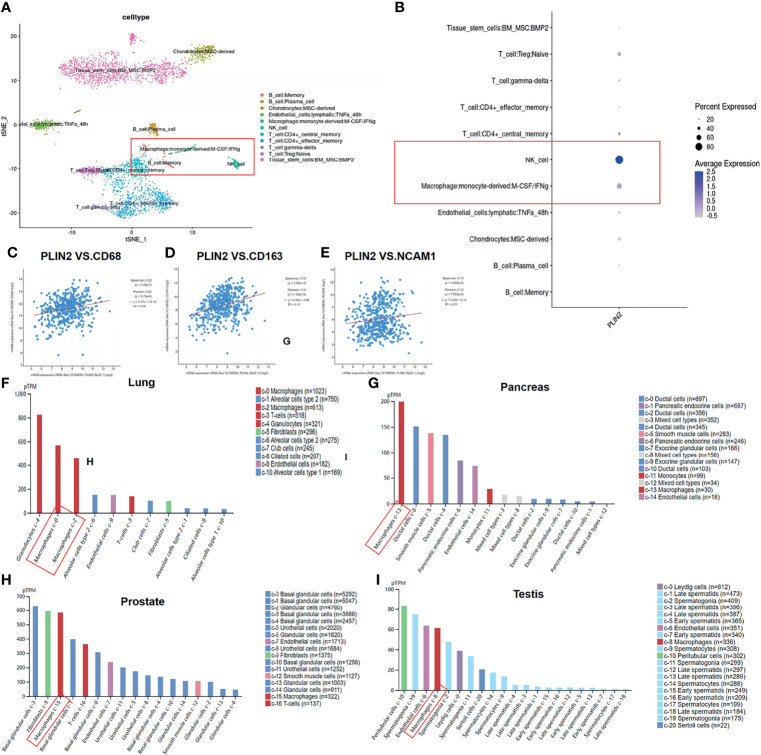
Expression pattern of *PLIN2* in OSCC was detected by single cell sequencing (GSE164690) **(A, B)**. The cBioPortal database was used to analyze the correlation between *PLIN2* and *CD68, CD163* and *NCAM1* in HNSCC **(C–E)**. Expression pattern of *PLIN2* in different tissues was analyzed using THE HUMAN PROTEIN ATLAS **(F–I)**.

Next, in order to figure out which type of immune cells these PLIN2-expressing cells belong to, we used single-cell sequencing (GSE164690) to analyze the expression pattern of PLIN2. UMAP PLOT visualized the cells in each cluster (**Figure 2A**), and the data showed that *PLIN2* was mainly expressed in NK cells, followed by tumor-associated macrophages (TAMs) in HNSCC (**Figure 2B**). At the same time, we used the cBioPortal database to analyze the correlation between *PLIN2* and macrophage surface markers *CD68*, *CD163*, and NK cell surface marker *NCAM1*; as shown by the data (**Figures 2C**–**E**), *PLIN2* was positively correlated with *CD68*, *CD163*, and *NCAM1*. Subsequently, we used THE HUMAN PROTEIN ATLAS (https://www.proteinatlas.org/ENSG00000147872-PLIN2/celltype) to search the expression pattern of *PLIN2* in different tissues and found that in the lung, pancreas, prostate, and testis, *PLIN2* was highly expressed in macrophages (**Figures 2F**–**I**). Therefore, we preliminarily speculated that *PLIN2* was highly upregulated in CD68^+^ TAMs in HNSCC and different tissues.

**Figure 3:**
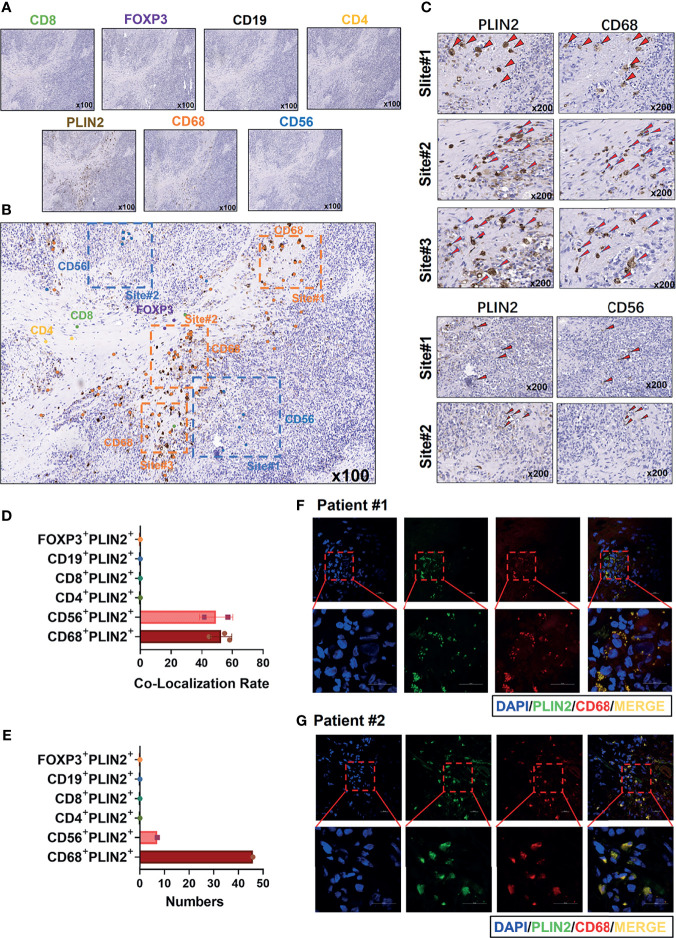
Immunohistochemistry was used to detect the coincidence degree of PLIN2 expression with CD4^+^T cell, CD8^+^ T cell, CD19^+^B cell, CD56^+^NK cells, CD68^+^TAMs, FOXP3^+^Tregs in OSCC serial sections **(A–C)**. The co-localization rate and numbers of CD4^+^PLIN2^+^, CD8^+^PLIN2^+^, CD19^+^PLIN2^+^, CD56^+^PLIN2^+^, CD68^+^PLIN2^+^, and FOXP3^+^PLIN2^+^ were analyzed **(D, E)**. Representative images of immunofluorescence staining from human OSCC sections using the PLIN2 antibody and CD68 antibody. The bar is 20 μm **(F, G)**.

Subsequently, we performed IHC staining of serial sections of PLIN2, CD4, CD8, CD19, CD56, CD68, and FOXP3 (**Figure 3A**). Moreover, we marked the main positive expression sites of different immune cell surface markers on the PLIN2 sections (**Figure 3B**). The results showed that PLIN2 and CD68^+^TAMs had a high co-localization rate, followed by CD56^+^NK cells. However, the number of CD56^+^PLIN2^+^ cells was far less than that of CD68^+^PLIN2^+^ cells, and there was no correlation with CD4, CD8, CD19 and FOXP3 (**Figures 3C**–**E**). The co-expression of PLIN2 and CD68 in TIIs was also observed with IF analysis (**Figures 3F, G**), suggesting that PLIN2 was strongly expressed in CD68^+^ TAMs in OSCC, followed by a small amount of expression in CD56^+^ NK cells.

### High PLIN2 Level in Tumor Center Predicts Advanced Stage of OSCC Patients

**Figure 4:**
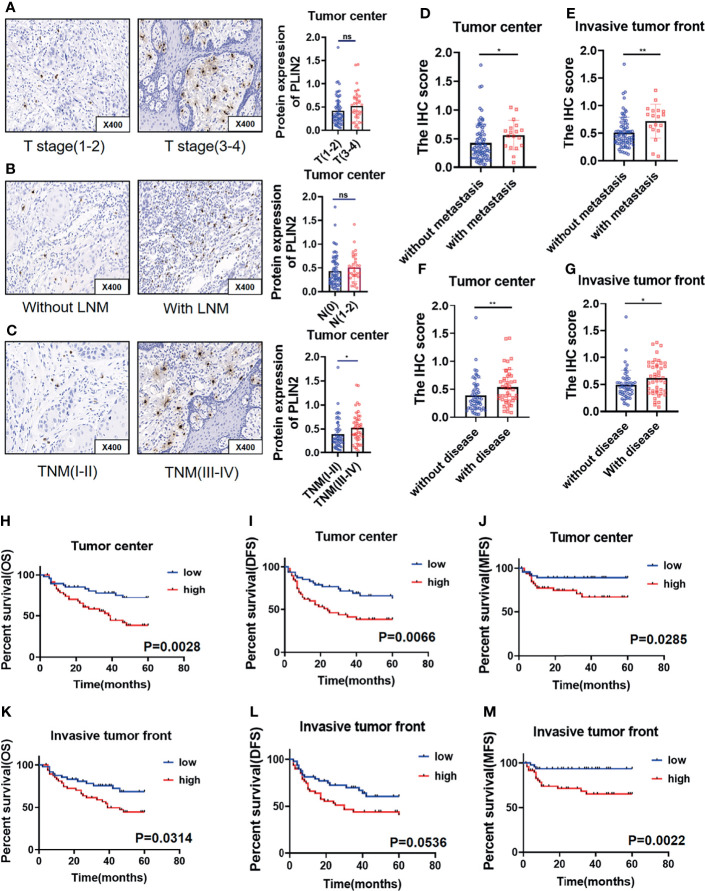
PLIN2 expression with different T stage **(A)** and lymph node metastasis **(B)** and TNM stage **(C)** in tumor center. Correlation between PLIN2 expression and metastasis status in tumor center and invasive tumor front **(D, E)**. Correlation between PLIN2 expression and disease status in tumor center and invasive tumor front **(F, G)**. Kaplan–Meier survival curves for overall survival time, disease-free survival time, and metastasis-free survival time of OSCC patients according to the expression of PLIN2 in tumor center **(H–J)** and invasive tumor front **(K–M)**. * and ** represented that differences were considered statistically significant with p < 0.05 and p < 0.01, respectively. ns represented no statistical differences.

We further analyzed the relationship between the expression of PLIN2 in tumor center and invasive tumor front with distinct clinicopathological variables, including sex, age, TNM, T stage, N stage, and differentiation. The results demonstrated that PLIN2 was significantly associated with TNM and T stage in tumor center (**Table 1**). However, PLIN2 with a high IHC score in OSCC patients was correlated with advanced TNM stage (p = 0.0112, **Figure 4C**), but not related to T stage and N stage (**Figures 4A, B**). According to these results, CD68^+^ TAM-derived PLIN2 might worsen the TNM stage to promote the OSCC malignant phenotype.

**Table 1:** Association between PLIN2 expression and clinicopathological characteristics in OSCC patients.

Characteristics	n = (96)	Tumor center		c^2^	*p*	Invasive tumor front		c^2^	*p*
		Low	High			Low	High		
**Sex**									
**Male**		26	23	0.375	0.540	26	23	0.375	0.540
**Female**		22	25			22	25		
**Age (years)**									
**<60**		20	21	0.043	0.837	24	17	2.086	0.149
**≥60**		28	27			24	31		
**TNM**									
**I–II**		32	17	9.379	0.002*^a^*	26	23	0.375	0.540
**III–IV**		16	31			22	25		
**T stage**									
**1–2**		37	25	6.558	0.01*^a^*	32	30	0.182	0.670
**3–4**		11	23			16	18		
**Lymph node metastasis**									
**No**		37	29	3.103	0.078	34	32	0.194	0.660
**Yes**		11	19			14	16		
**Differentiation**									
**Well**		17	17	0.000	1.000	15	19	0.729	0.393
**Moderate to poor**		31	31			33	29		
**Total**	96								

PLIN2, perilipin 2; OSCC, oral squamous cell carcinoma; TIIs, tumor-infiltrating immunocytes.

^a^Represented that differences were considered statistically significant with p < 0.05.

### Upregulated PLIN2 Correlated With Higher Risk of Postoperative Metastasis and Poor Survival

Considering that upregulated PLIN2 tends to have a malignant association with poor clinical outcomes, the association between PLIN2 and postoperative metastasis was further analyzed; postoperative disease status was also statistically analyzed, including postoperative metastasis and recurrence. The results showed that in the tumor center and invasive tumor front, the high expression of PLIN2 was associated with a higher postoperative metastasis rate (**Figures 4D, E**). Similarly, in the tumor center and invasive tumor front, high expression of PLIN2 tended to develop postoperative disease (**Figures 4F, G**). To confirm the prognostic value of PLIN2, Kaplan–Meier survival was used to analyze the survival of OSCC patients. The results showed that in the tumor center, patients with high PLIN2 expression had a shorter OS (**Figure 4H**, p = 0.0028), MFS (**Figure 4J**, p = 0.0285), and DFS (**Figure 4I**, p = 0.0066). In the invasive tumor front, the high expression of PLIN2 showed a shorter OS (**Figure 4K**, p = 0.0314) and MFS (**Figure 4M**, p = 0.0022), but there was no statistical difference in DFS (**Figure 4L**, p = 0.0536).

Univariate and multivariate Cox regression analyses were used to analyze the prognostic value of clinicopathological features. The results showed that sex, age, TNM, T stage, differentiation, and PLIN2 in the tumor center had no obvious prognostic value for OS and MFS. However, in OSCC, N stage was an independent prognostic indicator of OS and MFS. Moreover, the expression of PLIN2 in invasive tumor front was an independent prognostic indicator of MFS, not OS (**Table 2**). Taken together, these data indicated that PLIN2^+^CD68^+^TAMs were involved in tumor immune escape, which promoted the poor prognosis of patients.

**Table 2:** Prognostic factors in the Cox proportional hazards model for OS and MFS.

	OS					
Variables	HR	Univariate 95% CI	Sig.	HR	Multivariate 95% CI	Sig.
**Sex**						
**Female vs. male**	1.095	0.580–2.069	0.779			
**Age**						
**≥60 vs. <60**	1.110	0.583–2.113	0.752			
**TNM**						
**III–IV vs. I–II**	3.086	1.553–6.133	0.001*	0.929	0.314–2.753	0.895
**T-stage**						
**3–4 vs. 1–2**	1.684	0.888–3.194	0.111			
**Lymph node metastasis**						
**Yes vs. no**	4.394	2.298–8.403	<0.01*	4.048	1.510–10.848	0.005*
**Differentiation**						
**Moderate to poor vs. well**	1.765	0.872–3.571	0.114			
**PLIN2 in TIIs in tumor center**						
**High vs. low**	2.716	1.367–5.396	0.004*	1.748	0.805–3.795	0.158
**PLIN2 in TIIs in invasive front**						
**High vs. low**	2.045	1.046–3.997	0.037*	1.767	0.874–3.571	0.113

	MFS					
Variables	HR	Univariate 95% CI	Sig.	HR	Multivariate 95% CI	Sig.
**Sex**						
**Female vs. male**	1.364	0.538–3.458	0.513			
**Age**						
**≥60 vs. <60**	1.236	0.479–3.188	0.662			
**TNM**						
**III–IV vs. I–II**	10.841	2.486–47.280	0.002*	2.768	0.375–20.448	0.318
**T-stage**						
**3–4 vs. 1–2**	2.048	0.812–5.166	0.129			
**lymph node metastasis**						
**Yes vs. no**	11.275	3.676–34.579	<0.01*	6.335	1.404–28.593	0.016*
**Differentiation**						
**Moderate to poor vs. well**	2.199	0.723–6.692	0.165			
**PLIN2 in TIIs in tumor center**						
**High vs. low**	2.988	1.061–8.410	0.038*	0.958	0.309–2.971	0.941
**PLIN2 in TIIs in invasive front**						
**High vs. low**	5.508	1.594–19.035	0.007*	5.696	1.542–21.043	0.009*

TIIs, tumor-infiltrating immunocytes; PLIN2, perilipin 2; CI, confidence interval; OS, overall survival; MFS, metastasis free survival; HR, hazard ratio. * denoted that differences were considered statistically significant with p < 0.05.

### Dysregulated OSCC Immune Balance Accompanied by High PLIN2^+^CD68^+^ TAMs

**Figure 5:**
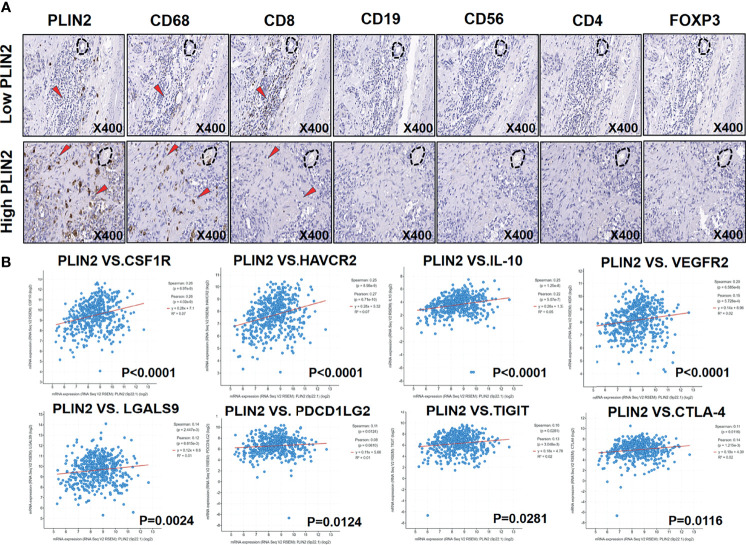
Serial immunohistochemical sections of OSCC followed by immunohistochemical examination were used to detect the relationship between PLIN2 expression level and CD4, CD8, CD19, CD56, CD68, and FOXP3 **(A)**. Correlation between *PLIN2* expression and *CSF1R*, *HAVCR2*, *IL-10*, *VEGFR2*, *LGALS9*, *PDCD1LG2*, *TIGIT*, and *CTLA-4* in HNSCC with cBioPortal database **(B)**.

Subsequently, we used serial sections to perform IHC staining of PLIN2, CD4, CD8, CD19, CD56, CD68, and FOXP3; the results showed that PLIN2 was inversely proportional to CD8^+^T cells and directly proportional to CD68^+^ TAMs and Foxp3^+^ Tregs (**Figure 5A**). Further, the TIMER database was also used for further analysis. **Table 3** shows the correlation between PLIN2 and specific immune cell marker infiltration at the transcription level. A partial correlation and a correlation adjusted by tumor purity were also provided. We found that PLIN2 was strongly related to important markers of various immune cells including CD8^+^ T cells, general T cells, B cells, monocytes, TAMs, M1 macrophages, M2 macrophages, neutrophils, natural killer cells, dendritic cells (DCs), Th1, Th2, follicular helper T cells, Th17, regulatory T cells, and T cell exhaustion. The results revealed that PLIN2 was strongly linked to immune infiltration in HNSCC, indicating the association between PLIN2 and immune imbalance in TME.

**Table 3:** Correlation analysis between PLIN2 and immune cell infiltrations in OSCC samples using TIMER.

Description	Gene markers	PLIN2			
		None		Purity	
		Cor	p	Cor	p
CD8^+^ T cell	CD8A	0.097	*	0.08	0.0762
	CD8B	0.152	***	0.135	**
T cell (general)	CD3D	0.099	*	0.083	0.0651
	CD3E	0.166	***	0.15	***
	CD2	0.165	***	0.148	**
B cell	CD19	0.158	***	0.15	***
	CD79A	0.119	**	0.112	*
Monocyte	CD86	0.347	****	0.337	****
	CSF1R	0.338	****	0.326	****
TAM	CCL2	0.326	****	0.314	****
	CD68	0.291	****	0.27	****
	IL10	0.317	****	0.311	****
M1 macrophage	NOS2	0.125	**	0.12	**
	IRF5	0.135	**	0.127	**
	PTGS2	0.014	0.757	0.033	0.466
M2 macrophage	CD163	0.382	****	0.374	****
	VSIG4	0.364	****	0.354	****
	MS4A4A	0.376	****	0.363	****
Neutrophils	CEACAM8	0.085	0.052	0.086	0.0563
	ITGAM	0.396	****	0.386	****
	CCR7	0.283	****	0.284	****
Natural killer cell	KIR2DL1	0.028	0.525	0.021	0.648
	KIR2DL3	0.08	0.0675	0.072	0.112
	KIR2DL4	-0.062	0.154	-0.061	0.178
	KIR3DL1	0.052	0.237	0.056	0.216
	KIR3DL2	0.16	***	0.15	***
	KIR3DL3	0.05	0.251	0.054	0.233
	KIR2DS4	0.085	0.0535	0.091	*
Dendritic cell	HLA-DPB1	0.215	****	0.196	****
	HLA-DQB1	0.172	****	0.158	***
	HLA-DRA	0.167	***	0.146	**
	HLA-DPA1	0.18	****	0.157	***
	CD1C	0.22	****	0.202	****
	NRP1	0.314	****	0.298	****
	ITGAX	0.383	****	0.384	****
Th1	TBX21	0.174	****	0.16	***
	STAT4	0.289	****	0.278	****
	STAT1	0.01	0.818	-0.017	0.705
	IFNG	-0.008	0.864	-0.026	0.564
	TNF	0.116	**	0.115	*
Th2	GATA3	0.194	****	0.184	****
	STAT6	0.051	0.243	0.044	0.335
	STAT5A	0.206	****	0.193	****
	IL13	0.19	****	0.175	****
Tfh	BCL6	0.243	****	0.248	****
Th17	STAT3	0.143	**	0.129	**
	IL17A	0.041	0.347	0.032	0.483
Treg	FOXP3	0.265	****	0.251	****
	CCR8	0.283	****	0.265	****
	STAT5B	0.367	****	0.352	****
	TGFB1	0.161	***	0.156	***
T cell exhaustion	PDCD1	0.112	*	0.096	*
	CTLA4	0.184	****	0.173	***
	LAG3	0.078	0.076	0.067	0.136
	HAVCR2	0.335	****	0.324	****
	GZMB	0.023	0.603	0.01	0.831

TIMER, Tumor Immune Estimation Resource; TAM, tumor-associated macrophage; Th, T helper cell; Tfh, Follicular helper T cell; Treg, regulatory T cell; Cor, R value of Spearman’s correlation; None, correlation without adjustment. Purity, correlation adjusted by purity. *，**，*** and **** represented that differences were considered statistically significant with P < 0.05, P < 0.01, P < 0.001 and P < 0.0001, respectively.

We found that PLIN2 was mainly expressed in CD68^+^ TAMs. Moreover, high PLIN2 levels in the invasive tumor front were related to a shorter MFS in patients with OSCC, and it was positively correlated with infiltrating CD8^+^T cell exhaustion. Thus, we speculated that PLIN2 might inhibit immune cell function to promote tumor metastasis. We further analyzed the correlation between PLIN2 and immune checkpoint molecules by cBioPortal. Interestingly, we found that *PLIN2* was positively linked to *colony stimulating factor 1 receptor* (*CSF1R* r = 0.26), *hepatitis A virus cellular receptor 2* (*HAVCR2* r = 0.25), *interleukin 10* (*IL-10* r = 0.25), *kinase insert domain receptor* (*VEGFR2* r = 0.20), *galectin 9* (*LGALS9* r = 0.14), *programmed cell death 1 ligand 2* (*PDCD1LG2* r = 0.11), *T cell immunoreceptor with Ig and ITIM domains* (*TIGIT* r = 0.10), and *cytotoxic T lymphocyte antigen 4* (*CTLA-4* r = 0.11) (**Figure 5B**). The results indicated that a high expression of PLIN2 might promote tumor immune escape by upregulating immune checkpoint molecules.

## Discussion

Currently, investigations into PLIN2 in several types of cancer have indicated that it has an important role in tumorigenesis. In Burkitt lymphoma, PLIN2 was mainly expressed in immature B lymphocytes (24). However, in colorectal cancer, lung adenocarcinoma, renal clear cell carcinoma, and pancreatic ductal adenocarcinoma, PLIN2 was mainly expressed in TCs (7, 9, 25–27). Notably, previous studies have demonstrated that in atherosclerotic lesions, PLIN2 was rich in macrophage-rich areas (28). Schmidt et al. have recently reported that PLIN2 could be expressed in adipocytes and several other cell types such as monocytes and macrophages (29). To sum up, the expression of PLIN2 in different tumors was heterogeneous. In this study, we also found that PLIN2 was mainly expressed in CD68^+^TAMs, and a small number of CD56^+^NK cells were also expressed. Therefore, we concluded that PLIN2 could participate in the occurrence and development of tumors by regulating CD68^+^TAMs in the TME.

Previous studies have suggested the relationship between PLIN2 or TAMs and tumor pathological parameters. As for PLIN2, in gastric cancer and aggressive melanoma (IM), PLIN2 overexpression cells were significantly associated with promoting cell proliferation and metastasis (11). Additionally, in IM, statistics showed overexpressing PLIN2 in the TNM stage III/IV than stage I/II (12, 30). As for TAMs, in OSCC, increased densities of CD68^+^, CD163^+^, and CD204^+^TAMs were significantly related to lymph node metastasis (31). Moreover, it was demonstrated that high infiltration of CD68^+^TAMs in tumor stroma (TS) was linked to a higher TNM stage in OSCC (32). Consistently, our results showed that the high expression of PLIN2 in the tumor center was related to advanced TNM stage, which featured as more infiltration of CD68^+^TAMs. The results suggested that PLIN2^+^CD68^+^TAMs may represent the tumor development degree in OSCC. Besides, we found that PLIN2 positively correlated with postoperative metastasis and disease. These results provided that PLIN2 may act as an oncogene in the tumorigenesis of OSCC.

The biological behavior of PLIN2 and TAMs seems to vary among tumors. On the one hand, high expression of PLIN2 was associated with poor MFS, DFS, and OS rates in cutaneous melanomas and related to poor prognosis in pancreatic ductal adenocarcinoma, breast cancer, and lung adenocarcinomas (12, 27, 33, 34). In contrast, other studies concluded that suppression of *PLIN2* promoted cell proliferation, invasion, and migration in uterine leiomyoma and renal cancer (13, 35, 36). On the other hand, studies have also revealed that high infiltration of CD68^+^ TAMs in TS was linked to a shorter OS or DFS (32). Consistently, our obtained data showed that upregulated PLIN2 correlated with a worse OS. Besides, Cox-regression analysis revealed that CD68^+^TAMs in TS were not an independent prognostic factor for OSCC patients (32). However, CD68^+^TAMs in the tumor nest (TN) were an independent prognostic marker for shorter OS and DFS in IBC (37). In this study, we found that PLIN2 in the invasive front was an independent risk factor for shorter MFS and CD68^+^TAMs were the main resource of PLIN2 in OSCC; thus, we speculated that the correlation between CD68^+^TAMs and outcome of tumor was distinct between TAM subsets or intratumoral distribution of TAMs (38). Indeed, data showed that the infiltration of the M2 subset and TAMs in TS was associated with poor prognosis, while the presence of the M1 subset and TAMs in TN was related to good prognosis in lung cancer (38). In summary, we identified that a high level of PLIN2^+^CD68^+^TAMs was an unfavorable indicator of survival time for OSCC patients, implicating that PLIN2 may be a promising therapeutic target. Moreover, further studies are needed to reveal the discrepant impact and underling molecular mechanisms of TAMs in the different interspaces of TME.

The presence of LDs was crucial for dendritic cell cross presentation to activate CD8^+^ T cells in cancer (15). LDs were also reported to harbor PGE2, which was a potent inhibitor of Th1 immunity and therefore responsible for alternative activation of macrophages (M2) to promote tumor growth and metastasis by suppressing tumor immune surveillance (39, 40). Furthermore, analysis of patients with colon cancer confirmed the positive correlation between the accumulation of LDs in TAMs and the clinical stage of tumor progression (40). Interestingly, PLIN2 was the most upregulated PLIN on LPS-LDs to integrate major intra- and extracellular immune responses (14). Our results also showed that high expression of PLIN2 in TME induced immunosuppression by inhibiting the activation of CD8^+^T cells and enhancing the capacity of CD68^+^TAMs and Foxp3^+^Tregs. Consistently, the results in TIMER showed that the expression of PLIN2 was positively correlated with macrophages, DCs, and tumor-infiltrating lymphocytes (TILs) in HNSCC.

In OSCC, low density of CD4^+^ FOXP3^+^ TILs was identified as an independent prognostic factor for poor OS and DFS (41). Also, the presence of high levels of CD8^+^ TILs correlated with longer OS (42). In squamous cell carcinoma of the oral tongue (OTSCC), Troiano et al. discovered that the immune-desert phenotype exhibited worse OS and DFS (43). However, in OSCC, we found that a high expression of PLIN2, which featured as less infiltration of CD8^+^ T cells and more Foxp3^+^ Tregs, predicted a worse OS and MFS, and it was an independent prognostic factor for MFS in the invasive front. Our findings preliminarily showed prognostic significance of specific TIL subpopulations in OSCC; the study of the immune phenotype and the molecular network needs to be further studied.

Considering the emergence of tumor drug resistance in immunotherapy, chemotherapy combined with immune-checkpoint inhibitor (ICI)-based immunotherapy has become an emerging strategy (44). In colon cancer, PLIN2 supported LD formation upon FOX and 5-Fu treatments; LD accumulation could consequently avoid PD-L1 and PD-1 exposure to tumor and CD8^+^T cells, respectively, thus leading to immunotherapy failure in addition to FOX resistance (45). Despite a great number of reports which highlighted the accumulation of PLIN2 in tumor progression, few studies have investigated the role of PLIN2 in tumor resistance to ICI.

Previous research has shown that high expression of HAVCR2, IL-10, and VEGFR2 reprogrammed TAMs and induced hepatocellular carcinoma (HCC), mouse breast cancer, and triple-negative breast cancer (TNBC) immunosuppression (46–48); targeting TAMs with an anti-CSF1R antibody improved T-cell checkpoint immunotherapy in pancreatic cancer models (49, 50). We also discovered that *PLIN2* expression positively correlated with *CSF1R*, *HAVCR2*, *IL-10*, *VEGFR2*, *LGALS9*, *PDCD1LG2*, *TIGIT*, and *CTLA-4* in HNSCC, indicating that the upregulated PLIN2 level in TME mediated ICI resistance. In pancreatic ductal adenocarcinoma (PDAC), LGALS9 polarized macrophages toward a M2 phenotype, leading to the inhibited secretion of T cell cytokines (51). This situation requires gaining a deeper understanding of the complex PLIN2 mechanisms and factors driving the activation of immune phenotype to clarify its role in mediating ICI resistance.

In conclusion, we identified that PLIN2 was associated with a higher risk of metastasis and PLIN2^+^CD68^+^TAMs can be used as a potential biomarker in the diagnosis and treatment of OSCC patients. Furthermore, PLIN2 was positively correlated with Foxp3^+^ Tregs and negatively correlated with CD8^+^ T cells, which indicated that its inhibited immune function was affected not only by TME but also through circulating TIIs. However, the molecular function and regulation pathway of PLIN2 in tumorigenesis of OSCC remained unexplored. In addition, the molecular mechanism of PLIN2 on immunomodulatory effects was still quite unclear. Future research is needed to unravel the role of PLIN2 in tumor progression through immunomodulation.

## Data Availability Statement

The original contributions presented in the study are included in the article/supplementary material. Further inquiries can be directed to the corresponding authors.

## Ethics Statement

All methods used for this study were approved by the Ethics Committee of Nanjing Stomatology Hospital (2019NL-009(KS)). The study was carried out in accordance with the Declaration of Helsinki. Written informed consent was obtained from all the patients.

## Author Contributions

Methodology, YH. Software, ZD. Validation, YD and XZ. Formal analysis, YS. Resources, XH and SC. Data curation, YH. Writing—original draft preparation, YH. Writing—review and editing, LD. Funding acquisition, ZW and YN. All authors contributed to the article and approved the submitted version.

## Funding

This work was supported by the National Natural Science Foundation of China (Grant Nos. 81902754, 81772880, 82002865, 81702680); Natural Science Foundation of Jiangsu Province (No. BK20190304); Fundamental Research Funds for the Central Universities (No. 021014380161); China Postdoctoral Science Foundation (No. 2019M651789); and Nanjing Medical Science and Technology Development Foundation, Nanjing Department of Health (Nos. YKK18123, YKK19091).

## Conflict of Interest

The authors declare that the research was conducted in the absence of any commercial or financial relationships that could be construed as a potential conflict of interest.

## Publisher’s Note

All claims expressed in this article are solely those of the authors and do not necessarily represent those of their affiliated organizations, or those of the publisher, the editors and the reviewers. Any product that may be evaluated in this article, or claim that may be made by its manufacturer, is not guaranteed or endorsed by the publisher.
